# Distributional potential of the *Triatoma brasiliensis* species complex at present and under scenarios of future climate conditions

**DOI:** 10.1186/1756-3305-7-238

**Published:** 2014-05-22

**Authors:** Jane Costa, L Lynnette Dornak, Carlos Eduardo Almeida, A Townsend Peterson

**Affiliations:** 1 Laboratório de Biodiversidade Entomológica, Instituto Oswaldo Cruz, Fundação Oswaldo Cruz, Rio de Janeiro, RJ, Brasil; 2 Biodiversity Institute and Departments of Ecology & Evolutionary Biology and Geography, University of Kansas, Lawrence, Kansas 66045, USA; 3 Departamento de Ciências Biológicas, Faculdade de Ciências Farmacêuticas (UNESP), Araraquara, SP, Brasil

**Keywords:** Chagas disease, Vectors, Predictions, Triatomines, Ecologic niche modeling, Biodiversity

## Abstract

**Background:**

The *Triatoma brasiliensis* complex is a monophyletic group, comprising three species, one of which includes two subspecific taxa, distributed across 12 Brazilian states, in the caatinga and cerrado biomes. Members of the complex are diverse in terms of epidemiological importance, morphology, biology, ecology, and genetics. *Triatoma b. brasiliensis* is the most disease-relevant member of the complex in terms of epidemiology, extensive distribution, broad feeding preferences, broad ecological distribution, and high rates of infection with *Trypanosoma cruzi*; consequently, it is considered the principal vector of Chagas disease in northeastern Brazil.

**Methods:**

We used ecological niche models to estimate potential distributions of all members of the complex, and evaluated the potential for suitable adjacent areas to be colonized; we also present first evaluations of potential for climate change-mediated distributional shifts. Models were developed using the GARP and Maxent algorithms.

**Results:**

Models for three members of the complex (*T. b. brasiliensis*, *N* = 332; *T. b. macromelasoma*, *N* = 35; and *T. juazeirensis*, *N* = 78) had significant distributional predictivity; however, models for *T. sherlocki* and *T. melanica*, both with very small sample sizes (*N* = 7), did not yield predictions that performed better than random. Model projections onto future-climate scenarios indicated little broad-scale potential for change in the potential distribution of the complex through 2050.

**Conclusions:**

This study suggests that *T. b. brasiliensis* is the member of the complex with the greatest distributional potential to colonize new areas: overall; however, the distribution of the complex appears relatively stable. These analyses offer key information to guide proactive monitoring and remediation activities to reduce risk of Chagas disease transmission.

## Background

Chagas disease affects millions of people across Latin America, yet no vaccine has been developed [1,2]. This disease is caused by a protozoan parasite, *Trypanosoma cruzi*, transmitted to humans mainly by blood-sucking bugs of the subfamily Triatominae [3]. Most triatomine species are distributed in Central and South America. Brazil holds the most diverse triatomine fauna, several of which are recognized as constituting complexes of species [4-6].

Multidisciplinary studies have indicated that the *Triatoma brasiliensis* complex is a monophyletic group [7-9] comprising four species, one of which includes two distinct subspecies, such that the relevant taxa in the group are *T. b. brasiliensis*, *T. b. macromelasoma*, *T. juazeirensis*, *T. melanica*, and *T. sherlocki*. Each member of the group can be identified by external morphological characteristics and a taxonomic key was recently published [9]. This complex is widespread in Brazil, occurring in 12 states, primarily within the caatinga and cerrado biomes [10]. Members of the complex present distinct epidemiological importance, morphological characteristics, natural history traits, ecological requirements, genetic characteristics, and dispersal abilities [11-19].

*Triatoma b. brasiliensis* is the best-studied member of the complex in terms of epidemiology and spatial distribution: it occurs in six states, uses diverse food sources, and is distributed across several ecotopes. Because this species presents one of *T. cruzi* the highest infection rates, as well as high intradomiciliary infestation rates, it is considered the most important Chagas disease vector in the region [6,10]. Despite significant reductions of Chagas disease incidence in Brazil in recent decades, *T. b. brasiliensis* and the parasites it transmits remain priority public health concerns [20,21]. However, the transitory nature of epidemiological importance is illustrated by the case of *T. sherlocki*, a species described as new to science only in 2002 [22], and subsequently recognized as a fifth member of the *T. brasiliensis* complex based on genetic and ecological characteristics [8,9]. Recently, however, this species was found colonizing human domiciles in a mining area in Bahia state, which highlights the quick changes that may occur in triatomine ecology, particularly in the face of drastic anthropogenic environmental changes [16]. Hence, monitoring vector capacity and epidemiological importance is crucial for understanding and controlling Chagas disease transmission [23,24].

One of the most important concerns regarding Chagas disease transmission is the degree to which vector species have the potential to colonize new areas, expanding risk areas for transmission. Some authors have suggested that degradation of sylvatic environments promotes invasion of new ecotopes by triatomines [25-27]. Another study, however, did not support the hypothesis that environmental degradation per se is the major factor in domiciliary invasions; instead, landscape patterns and characteristics of human dwellings may drive the probability that triatomines invade and colonize homes [28]. As several studies have shown that climatic characteristics shape geographic distributions of triatomines [29-32], tools that improve mapping of present-day triatomine distributions, as well as how they respond to climate change, are crucial to proactive measures in controlling and monitoring this disease. Costa and Peterson [33] recently reviewed ecological niche models (ENMs) as a tool permitting better understanding along these lines; and Diniz-Filho and colleagues [34] analyzed geographic patterns and environmental correlates of species richness in triatomine distributions.

ENMs offer a powerful approach to characterizing and differentiating ecological niches of vectors, broadening knowledge of relevant vector and parasite-reservoir distributions, defining areas at risk for transmission, and assessing overall potential geographic distributions of vector species. ENMs have been used to understand evolutionary processes involved in differentiating members of the *T. brasiliensis* complex [14], with patterns of ecological similarity matching patterns of molecular differentiation within the complex [7,11]. ENMs have also been useful in identifying vector-host associations [35] and mapping geographic distributions of triatomine species in detail [36,37]. In spite all this research attention, however, important distributional questions remain unaddressed.

Global climate change is anticipated to have critical impacts on biodiversity, with synergistic effects producing significant changes in distributions, natural history, and behavior of many organisms [38,39]. Climatic change has already influenced natural environments and human activities, and is likely already molding disease transmission [40-42]. ENMs have been used to make ‘forecasts’ of climate change effects on biodiversity [43]. Hence, here, we use ENM approaches (1) to develop estimates of potential geographic distributions for each member of the *T. brasiliensis* complex, and (2) to evaluate potential for colonizing new areas in response to climate change based on modeled future-climate scenarios for 2020 and 2050.

## Methods

### Occurrence points

Data were compiled from multiple sources: the entomological collections of Fundação Oswaldo Cruz, *secretarias de saúde* (health departaments) from states where the *T. brasiliensis* species complex occurs, and field collections by the research team of the Laboratório de Biodiversidade Entomológica - IOC - FIOCRUZ (2004–2008 [7,8,10,11,13,14,16]). In total, we assembled 459 occurrence points across 12 states on which to base ENMs for the complex; all specimens collected at these localities are deposited in the Entomological Collection of Instituto Oswaldo Cruz (CEIOC), spanning 1911–2008, including type specimens deposited by the authors of the species. All modern field work employed the same capture methods (i.e., capture by exhaustion).

Of the 459 occurrence points, most were readily identifiable based on external morphology and geographic range [9,10]. However, to confirm distributional limits for members of the complex, we selected 41 localities falling between ranges of different taxa, and made additional field captures of ~2160 specimens, all of which were identified carefully via reference to taxonomic studies [4,9,44-46], and by comparison with specimens in the collections of the Instituto Oswaldo Cruz, U.S. National Museum of Natural History, and American Museum of Natural History. As such, we can state confidently that identifications along these range borders were made correctly.

All records were plotted on maps, and checked further for consistency with species’ known ranges. *Triatoma b. brasiliensis* (*N* = 332), *T. b. macromelasoma* (*N* = 35), and *T. juazeirensis* (*N* = 78) had sufficient points for standard niche modeling techniques (described below); however, we had only limited occurrence data for *T. melanica* and *T. sherlocki* (each *N* = 7), which reflects their rarity and the scarcity of their populations. Occurrence data were divided randomly into calibration and evaluation data sets using 80% and 20% of points, respectively; because of small sample sizes for *T. melanica* and *T. sherlocki*, an alternative evaluation method was used (see Model evaluation, below). For models developed to evaluate implications of scenarios of future climates for the species complex as a whole, all occurrence data for the complex were combined into a single occurrence data set; this step maximizes sample sizes and avoids dispersal-limited situations in which distributional limits are set by environmental characteristics that are difficult to model [47].

### Environmental data

We used two sets of environmental data to construct models for each species. First, to estimate fine details of the current geographic distribution of each species, we used multitemporal Normalized Difference Vegetation Index (NDVI) from the Advanced Very High Resolution Radiometer (AVHRR; ~1 km native spatial resolution) sensor. These monthly NDVI images covered April 1992–March 1993. We chose imagery from this period because it best matched the bulk of our occurrence data (1992–2008). We assumed that NDVI patterns across the landscape in 1992–1993 was representative of the broader period.

Second, to develop coarse-resolution projections of the potential distributions of species under scenarios of global climate change for 2020 and 2050, we used the WorldClim data set at 1 km spatial resolution [48]. We included seven “bioclimatic” variables: annual mean temperature, mean diurnal temperature range, maximum temperature of the warmest month, minimum temperature of the coldest month, annual precipitation, and precipitation of the wettest and driest months [48]. Among the 19 available climatic variables within the Worldclim data set [49], these seven were relatively uncorrelated across the study region.

**Figure 1:**
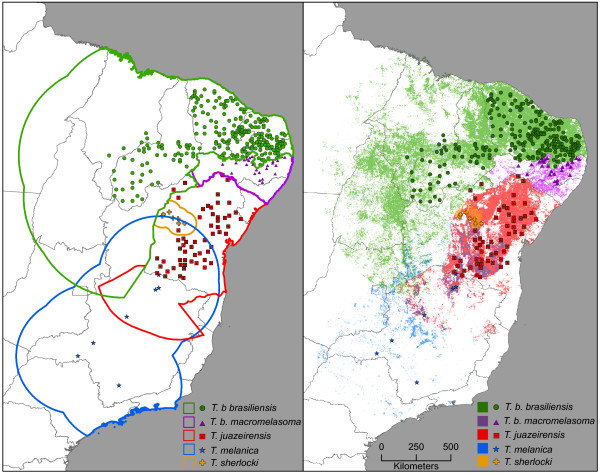
**Left: Map of hypothesized accessible areas (lines) and occurrence data (points) for all species in the *Triatoma brasiliensis *species group.** Right: Modeled current distributions for all members of the complex.

Following recent recommendations [50], we restricted model calibration to areas hypothesized to fall within the likely long-term movement and colonization potential (area **M**) of each species. To this end, for present-day distributional predictions of suitable habitat, we buffered occurrence points for each species by one half of the longest diameter of that species’ range. For climate change projections, we buffered around all points by a broader area of 500 km (i.e., combining species). After model calibration, major geographic features (e.g., São Francisco and Jequitinhonha rivers) were used to reduce M areas for each species, as members of this complex likely are not easily able to cross such barriers; we further considered neighboring distributions of species as barriers to dispersal (e.g., future zone between distributions of *T. b. brasiliensis* and *T. b. macromelasoma*; see summary in Figure 1).

### Model calibration

We used two algorithms and applied a consensus method to model ecological niches: a genetic algorithm (GARP [51-53]) and a maximum-entropy approach (Maxent [54]). Both algorithms relate environmental conditions to known occurrence records, and then compare those relationships to the landscape over which observations were derived (see detailed treatment in [55]). We combined the resulting models by mapping areas where predictions agreed from both algorithms. This final model can be used to identify areas presenting favorable environmental conditions across the landscape. Details of each approach follow.

GARP divides occurrence data into subsets for model calibration (rule training and intrinsic testing) and model evaluation (extrinsic testing). Through an iterative process, GARP uses calibration data to develop a set of rules from a variety of methods (BIOCLIM, logistic regression, etc.). GARP then modifies rules to maximize model predictivity, testing rules for accuracy, using the intrinsic testing data to determine whether rules should be incorporated into or rejected from the model. This process continues for 1000 iterations or until convergence, and models are evaluated using the extrinsic testing data [56]. We developed 100 model runs, with consensus via soft extrinsic omission thresholds [57]; all other parameters were left to default. The 10 “best” models were retained and summed to produce a final grid.

Maxent fits a probability distribution to species’ occurrences, such that entropy of the probability distribution is maximized (i.e., the probability distribution is maximally spread out), but subject to constraints imposed by the environmental characteristics of the occurrence data. Response curves (linear, quadratic, etc.) are constructed and then smoothed with regularization parameters to reduce model overfitting [58]. For Maxent models for estimating current distributions, we used default settings for all parameters, except that we selected a random seed. For climate change scenarios, we also used default settings, except that we specified a random seed with 50% of points set aside for model evaluation.

We used a minimum training threshold [59] to convert raw model outputs (i.e., continuous outputs) into actual distributional estimates (i.e., a binary representation). Such thresholds [59], also allowed us to scale Maxent and GARP models similarly. However, we modified this threshold to consider potential for error in the occurrence data. That is, instead of the threshold that includes 100% of the training data, we sought the highest threshold that includes (100 - *E*)% of the training data, where *E* is an estimate of the proportion of occurrence data that may include locational error [58]; we assumed *E* = 5%, based on our experience with these occurrence data.

### Model evaluation

In view of recent concerns [60] regarding robustness of the popular receiver operating characteristic (ROC) approach [61], we used a partial ROC area under the curve (AUC [62]) for *T. b. brasiliensis*, *T. b. macromelasoma*, and *T. juazeirensis*. This approach is based on traditional ROC approaches [63], but takes into account the degree of coverage of the commission error spectrum (*x*-axis) by model predictions, and prioritizes omission error over commission error in calculating model robustness. All tests were developed based on the random subsets of occurrence data described above. We used a Visual Basic program [64] to compare model AUCs to null expectations via bootstrapping 50% of the test points 1000 times, allowing for *E* = 5% error among the occurrence data.

For *T. melanica* and *T. sherlocki,* the partial ROC method was not appropriate because of small sample sizes (*N* = 7). Instead, we used a jackknife approach, designed for models based on limited occurrence data [64]. In this method, a set of models is built, each based on the occurrence but data omitting a different, single data point; these excluded points are used to test the predictive ability of the model, given a particular proportion of area predicted present [64]. For each algorithm and species, then, we constructed 7 models, each based on 6 training points, setting aside one point for model testing. All resulting models were thresholded as described above. These values and proportions of suitable areas for each model were used within the program provided by Pearson and colleagues [64].

### Ethics statement

Captures were carried out with technical support of technicians of the Secretaria de Vigilância em Saúde and Fundação Nacional de Saúde, which are the official governmental institutions charged with monitoring and controlling triatomines in domiciles in Brazil. All field collections followed established and approved procedures of those institutions, including obtaining permission from owners of domiciles.

## Results

In all, 459 occurrence sites for the *T. brasiliensis* species complex were included in the analysis. Taxonomic confirmations based in detailed comparison of color patterns matched with known geographic distributions of members of the complex [9,10]. In this sense, our data were in line with earlier results [8-10] indicating parapatric geographic distributions of members of the *T. brasiliensis* species complex, with the exception of marginal sympatry between *T. juazeirensis* and *T. melanica*[8], and between *T. juazeirensis* and *T. sherlocki*[17]. Figure 1 shows distributional data available for the five species of the complex, as well as the **M** hypotheses for each species.

Distributional estimates for the three species for which adequate sample sizes were available were statistically robust (*P <* 0.001). Predictive power of models for *T. melanica* and *T. sherlocki*, for which only minimal occurrence data were available, however, was not significant (all *P* > 0.05), using the Pearson small-sample test.

### Triatoma b. brasiliensis

Known occurrence data comes from the states of Maranhão, Piauí, Ceará, Rio Grande do Norte, and Paraíba, encompassing an extent of occurrence of about 1.6 × 10^6^ km^2^ (Figure 1). Model predictions included parts of those states, but also areas of Tocantins, Pará, Goiás, Minas Gerais, Distrito Federal, and Bahia, as falling within the species’ distributional potential. Occurrence of *T. b. brasiliensis* in Pernambuco is rare [19], although suitable conditions extend into this state. The southeastern edge of the predicted area for this species was restricted by the distribution of *T. b. macromelasoma*, which likely confines or obscures any possible dispersal potential in that direction.

### Triatoma b. macromelasoma

The distribution of this subspecies is limited to the state of Pernambuco [9,10], with an extent of occurrence of about 120,000 km^2^ (Figure 1). This state (and the species’ distributional area) is bordered by Piauí, Ceará, Rio Grande do Norte, and Paraíba (occupied by *T. b. brasiliensis*), and by Sergipe and Bahia (occupied by *T. juazeirensis*). These separations may be mediated by the São Francisco River and/or the presence of *T. juazeirensis* to the south; separation from *T. b. brasiliensis* depends on phenomena yet to be identified [14,19]. *Triatoma b. macromelasoma* is therefore constrained from population expansion on most sides by presence of other species, or via broadly unsuitable areas (to the north and southwest); however, within this defined region, a suitable area is restricted predominantly to the west of the Atlantic forest but east of the drier, more inland region of the caatinga (Figure 1).

### Triatoma juazeirensis

This species has been recorded almost exclusively in Bahia, in a broad area of about 500,000 km^2^ (Figure 1), with only a few records from southern Pernambuco [9,10]. The northern and western limits of its distributional area are set by the São Francisco River, and the southern limit may be set by the Jequitinhonha River. Suitable areas are limited almost completely to the caatinga region, with only minor extension into the cerrado in Minas Gerais or into the wetter Atlantic forest to the east. We note that two occurrences have been recorded north of the São Francisco River. These points may represent established resident populations; however, these bugs may have been transported there passively by humans, and may not be the result of natural colonization across the river (Figure 1).

### Triatoma melanica

Occurrence of this species is limited primarily to Minas Gerais, in an area of about 1.1 × 10^6^ km^2^ (Figure 1). However, it is known from one location in southern Bahia, where it is sympatric with *T. juazeirensis*. Because this species is known from only seven sites, model accuracy was poor, and predictions regarding its distribution should be considered with caution. Our models show sparse areas of suitability across seven states: Bahia, Distrito Federal, Goiás, Minas Gerais, Mato Grosso do Sul, São Paulo, and Rio de Janeiro. In Bahia, areas possibly suitable for *T. melanica* overlap *T. juazeirensis* east of the São Francisco River (Figure 1).

### Triatoma sherlocki

Records for this species are limited to north-central Bahia, in a circumscribed area of about 40,000 km^2^ (Figure 1). *Triatoma sherlocki* is the most restricted member in the group as regards potential distributional area, and appears to be confined to the northern part of the Chapada Diamantina. It remains unclear whether *T. sherlocki* and *T. juazeirensis* are parapatric or sympatric, because both occur under the same general conditions (cracks, crevices, and holes in exposed rocks) within the caatinga. However, the area of transition between the two remains unknown, awaiting in-depth, on-the-ground surveys. Model predictions were poor for this species, so interpretation of results should be made with caution (Figure 1).

### Climate change scenarios

**Figure 2:**
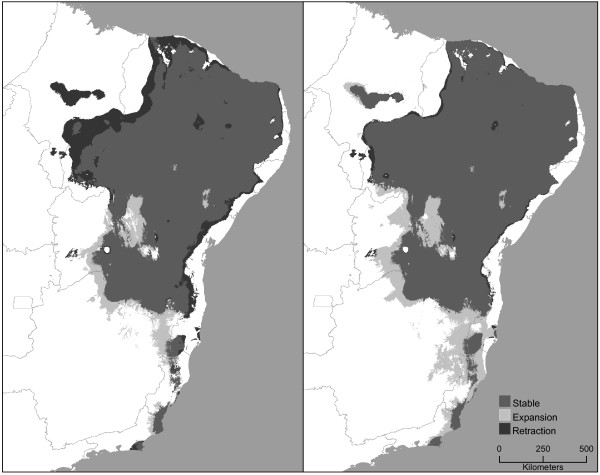
Climate change scenarios for the T. brasiliensis species complex in for 2020 (left) and 2050 (right), showing stable areas (medium gray) and areas of potential expansion (light gray) and potential retraction (dark gray).

Within the distribution of the *T. brasiliensis* complex, the climate change scenario explored here suggests an increase in mean temperature of 1.72°C and decrease in mean precipitation of 55.6 mm by 2050 across the region. Projections of niche model rule sets onto future climate conditions for the complex suggested little likely change in species’ distributions with changing climates (Figure 2). Near-term projections (2020) anticipate areas of retraction in the northwest, along the western edge of the distribution of *T. b. brasiliensis*, as well as along the eastern margin where the cerrado meets Atlantic forest. Some possible expansion may be expected in central Bahia and northern Minas Gerais. Overall, however, the distribution of the complex remains largely stable under these projections. The longer-term projections (2050) yielded more or less similar patterns, with expansion anticipated in the same areas, but without retraction (Figure 2).

Additional file 1 shows biomes and political boundaries of Brazilian states across the distribution at areas of members of the *T. brasiliensis* species complex.

## Discussion

### General comments

This study presents a first comprehensive round of niche modeling and mapping of potential distributions for members of the *T. brasiliensis* complex, although several of our previous studies have touched on similar topics [14,33,35,37]. It worth mentioning that our assumption that neighboring distributions may serve as barriers to dispersal was based on background information posed of periodic field work since 1996 [7,8,10,11,13,14,16]. Until now, we have never collected two species in the same location. Species distribution seems to have remained stable until now. Given our knowledge of these species and their dispersal capacity, we show all the potential areas that are suitable given the restrictions to the dispersal capacity of each. The general approach and reasoning are also presented in greater detail by authors [50]. Model predictions for *T. b. brasiliensis*, *T. b. macromelasoma*, and *T. juazeirensis* were statistically significantly robust in their distributional predictions; however, models for *T. sherlocki* and *T. melanica* were unable to make predictions that were better than random. Because the five members of the complex show distinct epidemiologic importance [10,13,16] and dispersal abilities [17], these distributional patterns have considerable importance for human exposure patterns.

For example, *T. melanica* is a highly differentiated member of the complex [7,9,11,12], and occupies sylvatic ecotopes, invading homes only sporadically; indeed, we know of no records of broad domiciliary colonization by this species [10,45]. On the other hand, *T. sherlocki*, the most distinct species on all grounds [8,10] described recently as a sylvatic species new to science [22], was recently found colonizing domiciles constructed relatively near natural rock piles in a mining area [16,17]. Models for both of these species identified only restricted suitable areas, yet the epidemiological potential in real life appears to differ markedly.

Despite the fact that *T. sherlocki* occurs in areas close to the distribution of *T. juazeirensis*, it may occupy distinct microhabitats among rocky outcrops [16,46]. *Triatoma sherlocki* is the only species in the complex that is flightless, although its dispersal capacity may be compensated by longer legs and possibly better walking ability [17]. A recent study found support for high reproductive compatibility between *T. sherlocki* and the remaining members of *T. brasiliensis* species complex [18]. What is more, laboratory progeny between the parapatric *T. juazeirensis* and *T. sherlocki* were more vigorous in terms of longevity and dispersal capacity [17,65]. However, despite the physical proximity and genetic compatibility of these two species, no hybrids or intermediate forms have yet been collected in the field, such that factors involved in their isolation remain unknown [17-19]. Recent experiments under laboratory conditions showed that *T. juazeirensis* presents higher reproductive potential as regards fecundity and fertility when compared to *T. sherlocki*[65].

*Triatoma juazeirensis* is restricted almost entirely to Bahia, although a few specimens with this external phenotype have been collected recently in Pernambuco. These occurrences may represent genetic reassortments in a hybrid zone between *T. juazeirensis* and *T. b. macromelasoma*[19], leading to elevated phenotypic variability in Pernambuco. The alternative hypothesis is that *T. juazeirensis* phenotypes reflect recent dispersal of the species into new areas.

Our models suggest that the member of the complex with greatest opportunity to colonize new areas is *T. b. brasiliensis*. Model projections suggest that it could find suitable conditions in the central part of the cerrado, in Tocantins and Goiás, northwestern Minas Gerais, and western Bahia. This species is known to have high genetic variability [7] and broad feeding habits [13,66], such that it may be able to explore new environmental and ecological possibilities [67]. Therefore, the areas mentioned above should be monitored for colonization by *T. b. brasiliensis*. In fact, a few records for this species state have already been accumulated in Tocantins by technicians of the Fundação Nacional de Saúde/Secretaria de Saúde [10].

### Climate and environmental changes

Models that estimate climate change effects on species’ distributions can be important tools in anticipating distributional shifts [68]. Triatomines may be able to respond rapidly to environmental changes, which may in turn influence their biological cycles. The effects of climate on triatomines and *T. cruzi* transmission have been studied almost since the original description of Chagas disease: in *T. infestans*, warmer temperatures both accelerate embryonic development [69] and allow for additional generations per year [70]. Therefore, anticipation of distributional shifts with respect to future climate scenarios is essential for effective control of Chagas disease transmission.

The *T. brasiliensis* complex is distributed chiefly within two biomes [9,10,45,46], in habitats that are anticipated to see rather strong climate change effects, at least on vegetation [71]. Indeed, beyond simple distributional effects that are the object of the niche models, increasing temperatures may have more direct physiological effects on triatomines, such as elevated metabolic rates, elevated egg production, etc. [72-77]. Such shifts could have important impacts on vectorial transmission of Chagas disease by members of the complex. Nonetheless, the distributional consequences anticipated by our models appear to be subtle or nearly negligible, suggesting that this complex in this region is unlikely to show large-scale distributional shifts in reaction to changing climates.

### Special considerations

Several factors potentially may have affected the models that we have explored. A first comment concerns *T. melanica* and *T. sherlocki*. First, five of the seven occurrence points for *T. melanica* were collected within small towns and cities; however, the satellite products (NDVI) we used measured greenness of the landscape. In this case, then, the algorithms were likely relating the data points to reflectance patterns characteristic of urban areas. Although it is important to identify all potential habitats (including urban environments), we do not feel that our environmental data permitted our models to characterize the sylvatic environment for this species adequately. In larger data sets, as for *T. b. brasiliensis* or *T. juazeirensis*, urban-collected points would be joined by others that give a clearer view of all environmental conditions used by the species.

Second, four data points for *T. sherlocki* were georeferenced precisely, but perhaps incorrectly, as falling in areas uncharacteristic of their natural environments (e.g., in the center of agricultural fields away from large rock outcrops). It is possible that these samples represent coarse-spatial-resolution representations of otherwise correct locations, or passive human transport; regardless, these points probably do not correspond to established colonies, consequently affecting model results. Clearly, further documentation of the distributional ecology of this species is needed.

Third, both *T. melanica*[9,10,45] and *T. sherlocki*[16,17] are essentially sylvatic (the domiciliation process for *T. sherlocki* may be a new event [6]). For the latter species, the proximity of its natural ecotope (rocky outcrops) and human domiciles (also sometimes built with rocks), combined with sylvatic hosts might have pressured this change in behavior, as has been suggested previously for other triatomines [67]. Our data for these species may have been biased geographically because most records came from long-term (~20 years) surveys by the Secretaria de Saúde, which only surveys human domiciles. Broader searches in sylvatic environments would likely increase numbers of occurrence points for these species, and would therefore improve model predictions in the future.

One final concern relates to the two *T. juazeirensis* points from north of the São Francisco River. Passive transportation of bugs has been implicated in dispersal of *T. infestans* across the Southern Cone of Latin America. After domiciliation, insects can easily be transported by humans (e.g., in clothes or luggage [78,79]). We suggest that *T. juazeirensis* occurrences north of the São Francisco River may be attributed to such human-mediated passive transportation. Other possible explanations for these occurrence points include misidentification, poor georeferencing, or unexpected phenotypes generated by hybridization and reassortment [19]. Regardless, this river likely functions at least as a partial natural barrier to northward expansion of this species, since records north of the river are very uncommon.

## Conclusions

Chagas disease affects millions of people across Latin America, yet this disease is still largely neglected. In eastern Brazil, the *T. brasiliensis* species complex represents the group of vectors with the greatest potential to transmit this disease. Until now, the extent of the potential distribution of habitat for each member of this complex was not fully characterized. Our results indicated that, of all members of this complex, *T. b. brasiliensis* exhibits the greatest potential to colonize new areas; however, our model projections do not anticipate much change in the distribution of the complex as a consequence of climate change. Hence, here, we present information useful in guiding proactive monitoring and remediation activities to reduce risk of further Chagas disease transmission in northeastern Brazil.

## Competing interests

The authors declare that they have no competing interests.

## Authors’ contributions

Conceived the study: JC and ATP. Collected data: JC and CEA. Identified samples: JC; Model and figure production: LLD and ATP. Wrote the manuscript: JC, LLD, CEA, and ATP. All authors read and approved the final version of the manuscript.

## Supplementary Material

Additional file 1 **Map showing biomes and political boundaries of Brazilian states across the distribution at areas of members of the *Triatoma brasiliensis *** **species complex.** AL = Alagoas, AP = Amapá, BA = Bahia, CE = Ceará, DF = Distrito Federal, ES = Espírito Santo, GO = Goiás, MA = Maranhão, MT = Mato Grosso, MS = Mato Grosso do Sul, MG = Minas Gerais, PR = Paraná, PB = Paraíba, PA = Pará, PE = Pernambuco, PI = Piauí, RJ = Rio de Janeiro, RN = Rio Grande do Norte, SE = Sergipe, SP = São Paulo, TO = Tocantins. Click here for file
