# Supplementary figures and images for: Distributional potential of the Triatoma brasiliensis species complex at present and under scenarios of future climate conditions
Source: Parasit Vectors. 2014 May 22;7:238. doi: 10.1186/1756-3305-7-238 (PMC4046994; doi:10.1186/1756-3305-7-238)

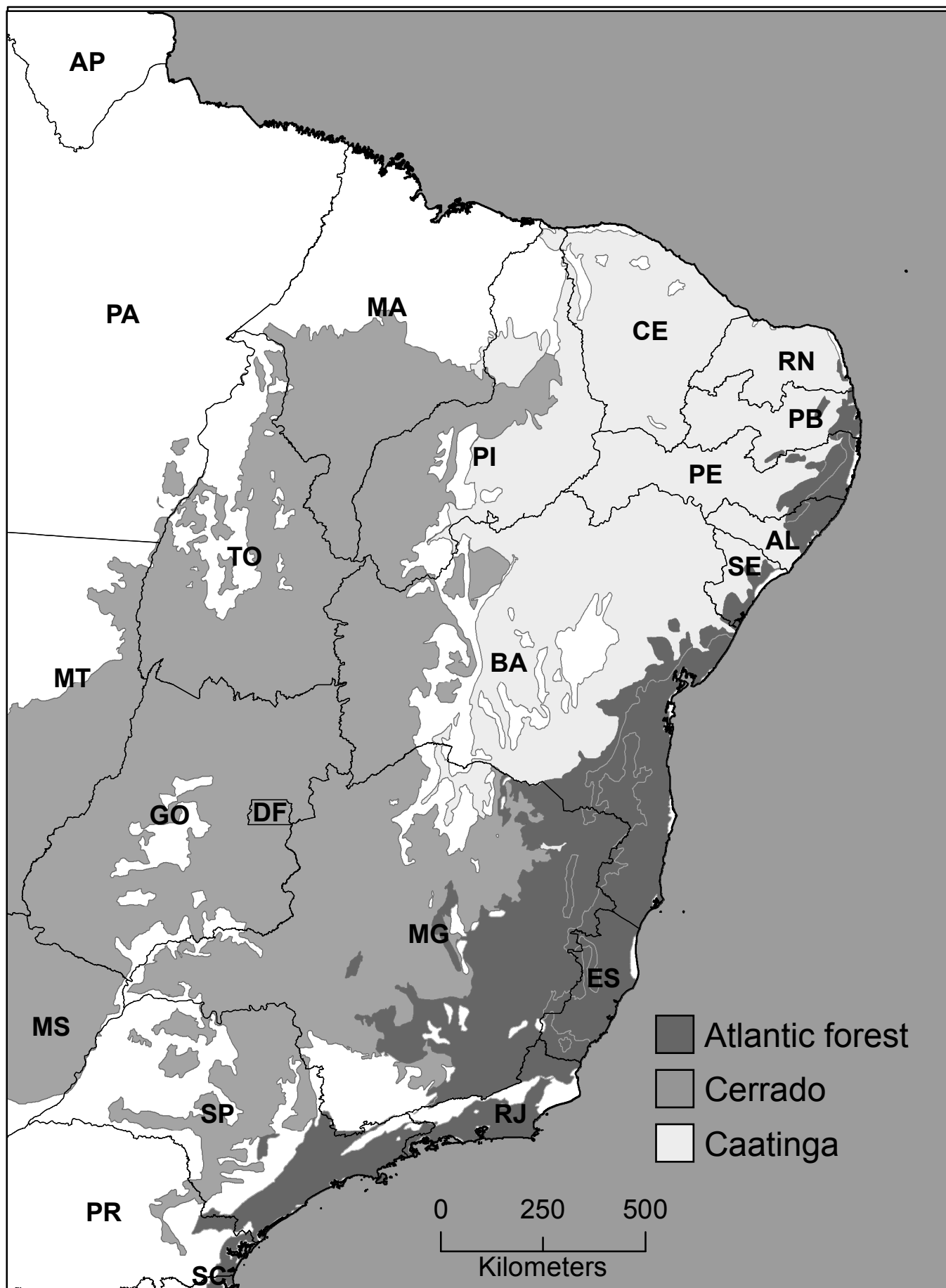

Supplement: Additional file 1 — Map showing biomes and political boundaries of Brazilian states across the distribution at areas of members of the Triatoma brasiliensis species complex. AL = Alagoas, AP = Amapá, BA = Bahia, CE = Ceará, DF = Distrito Federal, ES = Espírito Santo, GO = Goiás, MA = Maranhão, MT = Mato Grosso, MS = Mato Grosso do Sul, MG = Minas Gerais, PR = Paraná, PB = Paraíba, PA = Pará, PE = Pernambuco, PI = Piauí, RJ = Rio de Janeiro, RN = Rio Grande do Norte, SE = Sergipe, SP = São Paulo, TO = Tocantins. [file 1756-3305-7-238-S1.pdf]
